# Large Electrocaloric Effect in Relaxor Ferroelectric and Antiferroelectric Lanthanum Doped Lead Zirconate Titanate Ceramics

**DOI:** 10.1038/srep45335

**Published:** 2017-03-27

**Authors:** Biao Lu, Peilian Li, Zhenhua Tang, Yingbang Yao, Xingsen Gao, Wolfgang Kleemann, Sheng-Guo Lu

**Affiliations:** 1 Guangdong Provincial Key Laboratory of Functional Soft Condensed Matter, School of Materials and Energy, Guangdong University of Technology, Guangzhou, 510006, China; 2 Institute for Advanced Materials and Guangdong Provincial Key Laboratory of Quantum Engineering and Quantum Materials, South China Normal University, Guangzhou, 510006, China; 3 Angewandte Physik, Universität Duisburg-Essen, D-47048, Duisburg, Germany

## Abstract

Both relaxor ferroelectric and antiferroelectric materials can individually demonstrate large electrocaloric effects (ECE). However, in order to further enhance the ECE it is crucial to find a material system, which can exhibit simultaneously both relaxor ferroelectric and antiferroelectric properties, or easily convert from one into another in terms of the compositional tailoring. Here we report on a system, in which the structure can readily change from antiferroelectric into relaxor ferroelectric and vice versa. To this end relaxor ferroelectric Pb_0.89_La_0.11_(Zr_0.7_Ti_0.3_)_0.9725_O_3_ and antiferroelectric Pb_0.93_La_0.07_(Zr_0.82_Ti_0.18_)_0.9825_O_3_ ceramics were designed near the antiferroelectric-ferroelectric phase boundary line in the La_2_O_3_-PbZrO_3_-PbTiO_3_ phase diagram. Conventional solid state reaction processing was used to prepare the two compositions. The ECE properties were deduced from Maxwell relations and Landau-Ginzburg-Devonshire (LGD) phenomenological theory, respectively, and also directly controlled by a computer and measured by thermometry. Large electrocaloric efficiencies were obtained and comparable with the results calculated via the phenomenological theory. Results show great potential in achieving large cooling power as refrigerants.

Cooling technologies have been widely used in industry, agriculture and human daily life. Vapor compression plays a principal role in current cooling technologies and is still the core component of refrigeration systems. However, the coolant used in the vapor compressors are usually not environmentally friendly^1^. Besides, vapor compression is less efficient than solid state cooling^2^.

Several promising cooling technologies are currently under research and development, such as solar sorption, thermoelectric, magnetocaloric and electrocaloric cooling^3^. Among these cooling technologies, the electrocaloric one enjoys the most advantages^3^. The electrocaloric effect (ECE) is the adiabatic temperature change or isothermal entropy change caused by a polarization variation of a polar material upon the application or withdrawing of an external electric field^4^,^5^. Theoretical simulation indicates that the coefficients of performance (COPs) of the cooling device based on ECE can achieve more than sixty percent of the Carnot efficiency, which is much larger than that of the vapor compressor^6^.

When an electric field is applied to the electrocaloric material, the electric dipoles become ordered from a disordered state, which leads to the reduction of entropy associated with the polarization. Since the total entropy of the material remains constant, the entropy of the lattice will be increased to compensate the reduction of dipolar entropy under adiabatic conditions. Thus the temperature of the ECE material will be increased. When the external electric field is removed, however, the entropy of the electric dipoles increases due to the transition from an ordered to a disordered state, the temperature of the ECE material will be reduced under adiabatic conditions^4^,^5^,^7^. Based on above analyses, strongly polar materials, such as ferroelectrics (FE) and antiferroelectrics (AFE), will be promising ECE candidates^7^, since the larger the polarization change the larger the ECE will be.

Relaxor ferroelectrics possess a glassy polar phase^8^,^9^, in which nanosized polar domains are distributed randomly throughout the volume of the material. Thus, the multiple possible orientations of the polar domains might generate an enhanced ECE according to recent calculation^10^. On the other hand, an antiferroelectric to ferroelectric phase transition will be induced when a large enough electric field is applied. During this process, the reorientation of the two opposite dipoles in a unit cell may lead to a large entropy change. In addition, a significant ECE is usually associated with the phase transition. Since antiferroelectrics have more types of phase transition (i.e., AFE-FE phase transition) than their ferroelectric counterparts, they are also likely to have higher ECEs^7^.

In this study, two PLZT compositions (Pb_0.89_La_0.11_(Zr_0.7_Ti_0.3_)_0.9725_O_3_ and Pb_0.93_La_0.07_(Zr_0.82_Ti_0.18_)_0.9825_O_3_) were designed in the multiphase region, which are near to the relaxor ferroelectric and antiferroelectric regions (see Fig. 1). The PLZT ceramics based on the two compositions were prepared by a conventional solid state reaction process. The ECE of the ceramics were then investigated by using Maxwell relations and Landau-Ginzburg-Devonshire (LGD) phenomenological theory in the temperature range of 293 to 423 K. However, the phenomenological theories often refer to ideal conditions such as single domain states and ergodic conditions^3^. Hence, for polycrystalline ceramics the ECE deduced by idealized phenomenological theories may not be consistent with the experimental facts^11^. In order to estimate the deviations, the ECEs were also directly electric field controlled by a computer and measured by thermometry. Finally, the predictions deduced from Maxwell relation and phenomenological LGD theory are compared to the results obtained by the direct measurements.

## Results

### Microstructure and phase composition

The microstructures of the PLZT ceramics are illustrated in Figure S1 in the Supplementary Information. From the SEM images dense morphologies of both samples can be inferred. As proved by the XRD patterns (Figure S2 in the Supplementary Information), no excess PbO peak appears in the two compositions^12^. It was suggested that the addition of La^3+^ ions produces a significant number of lattice vacancies and results in the enhancement of the densification in the PLZT ceramics^13^. After the sintering process as shown in Table 1, the average grain size of Pb_0.93_La_0.07_(Zr_0.82_Ti_0.18_)_0.9825_O_3_ is in the range of 1~3 μm, while that of Pb_0.89_La_0.11_(Zr_0.7_Ti_0.3_)_0.9725_O_3_ in 0.5~2 μm. Furthermore, the grain sizes of the sintered ceramics are basically uniform. Ceramics with fine grains and uniform grain sizes usually possess higher breakdown field strength^14^.

The XRD patterns of the PLZT ceramics at room temperature are shown in Figure S2 in the Supplementary Information. The peaks of the samples are consistent with the standard XRD pattern of the polycrystalline perovskite structure^15^. Besides, no impurity phase can be detected in the pattern. Figure S2 present the highlighted XRD patterns for (1 1 0) (around 31°), (2 0 0) (around 44°) and (2 2 2) peaks (around 82°). According to Figure S2, it can be concluded that the XRD pattern of Pb_0.89_La_0.11_(Zr_0.7_Ti_0.3_)_0.9725_O_3_ belongs to a cubic phase. However, the (2 0 0) peak is broader than the (1 1 1) peak. This phenomenon might be caused by the spontaneous polarization in the crystalline phase. The hysteresis loop (Fig. 2(a)) also supports the existence of polarization. Thus, the crystalline phase of the Pb_0.89_La_0.11_(Zr_0.7_Ti_0.3_)_0.9725_O_3_ seems a pseudo-cubic structure^16^.

For the Pb_0.93_La_0.07_(Zr_0.82_Ti_0.18_)_0.9825_O_3_ sample, the position of each peak shifts toward lower diffraction angles. Double (200) peaks and double (222) peaks are observed in the XRD patterns, which indicate the coexistence of both rhombohedral and orthorhombic phases in these compositions.

### Dielectric property and relaxation behavior

The temperature dependences of permittivity and dielectric loss tangent for each sample are depicted in Figure S3(a) and (b) in the Supplementary Information. The permittivity and loss tangent as functions of temperature for sintered samples without any aging process, were measured in the heating run. As shown in Figure S3(a), the permittivities as a function of temperature at three frequencies (1, 10 and 100 kHz) show broad peaks covering the whole temperature range. When the frequency increases, the peak temperatures of the permittivity of Pb_0.89_La_0.11_(Zr_0.7_Ti_0.3_)_0.9725_O_3_ shift towards higher temperatures and the loss tangents become larger. These two features are typical characteristics of relaxor ferroelectrics^17^,^18^. By contrast, the permittivity of Pb_0.93_La_0.07_(Zr_0.82_Ti_0.18_)_0.9825_O_3_, as shown in Figure S3(b), does not show obvious dielectric characteristic of relaxor ferroelectrics. The Curie temperature of Pb_0.93_La_0.07_(Zr_0.82_Ti_0.18_)_0.9825_O_3_ remains almost constant as the frequency increases. Besides, the modified Curie-Weiss law was also employed to further describe the relaxation behavior of the two samples and the specific details are listed in the Supplementary Information. The results indicate that the two samples show typical relaxor ferroelectric behaviors. The apparent Curie temperatures are about 330 K for Pb_0.89_La_0.11_(Zr_0.7_Ti_0.3_)_0.9725_O_3_ and 405 K for Pb_0.93_La_0.07_(Zr_0.82_Ti_0.18_)_0.9825_O_3_. Based on previous studies, the Curie temperature of PLZT system is mainly affected by the lanthanum content, where it usually decreases with increasing lanthanum content^19^.

### ECE calculation and measurement

In order to calculate the ECE of the PLZT ceramics, the polarization – electric field (P–E) hysteresis loops at 10 Hz were measured at an increment of 10 K in the temperature range of 293 K to 423 K. The P–E hysteresis loops are presented in Fig. 2(a) and (b). As shown in Fig. 2(a), slim hysteresis loops, which are characteristic of relaxor ferroelectrics^20^, can be observed at each testing temperature. Although the nominal composition of Pb_0.89_La_0.11_(Zr_0.7_Ti_0.3_)_0.9725_O_3_ is located at the border of antiferroelectric and relaxor ferroelectric phases, our sample merely demonstrates features of relaxor ferroelectrics. The P–E hysteresis loops of Pb_0.93_La_0.07_(Zr_0.82_Ti_0.18_)_0.9825_O_3_ (Fig. 2(b)), whose composition is located in the triangle area of antiferroelectric, normal ferroelectric and relaxor ferroelectric phases, show double hysteresis loops with an open gap at the origin below 403 K, which is mainly caused by the coexistence of antiferroelectric and a small amount of ferroelectric phases^20^. When the temperature is above 403 K, as shown in Fig. 2(b), ferroelectric hysteresis loops can be observed. This phenomenon is attributed to the temperature induced phase transition of antiferroelectric to ferroelectric phase at about 403 K.

In the Supplementary Information, Figure S4(a) and (b) show the polarization as a function of temperature for the two samples. The polarization at various temperatures and electric fields has been extracted from the upper branches of the P–E hysteresis loops at E > 0 as presented in Fig. 2(a) and (b). Then the polarization as a function of temperature, *P(T*)_*E*_, was obtained from an 8th-order polynomial fitting to the cubic-spline interpolation of the raw *P(T*)_*E*_ data. For the relaxor ferroelectric, the polarization decreases with increasing temperature at any external electric fields (E ≥ 0), as shown in Fig. 2(a) and S4(a). The polarization of the antiferroelectric ceramic differs appreciably from that of the relaxor ferroelectric. According to Fig. 2(b) and S4(b), the polarization at external electric fields *E* = 0–3.5 MV/m decreases with the increasing temperature below ~400 K, but increases within *T* = 400–423 K due to the transition from the antiferroelectric to the ferroelectric phase. When the external electric field exceeds 3.5 MV/m, the polarization decreases with increasing temperature in the whole temperature range of *T* = 293–423 K.

Based on the Maxwell relation 
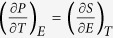
, reversible adiabatic changes in entropy (*ΔS*) and temperature (*ΔT*) are approximately given by^1^,^4^,^5^


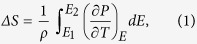



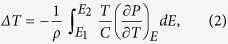


where *ρ* and *C* are the bulk density and specific heat capacity of the ceramics. The *E*_*1*_ and *E*_*2*_ denote the start and end electric fields. For normal ferroelectrics and relaxor ferroelectrics, *E*_*1*_ is equal to 0^21^,^22^,^23^. However, for antiferroelectric materials, *E*_*1*_ is the limit, above which the antiferroelectric regime is avoided and 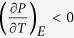 is ensured^24^,^25^. The upper integration limit *E*_*2*_ is the maximum field applied^25^. As analyzed above, we find *E*_*1*_ = 3.5 MV/m for Pb_0.93_La_0.07_(Zr_0.82_Ti_0.18_)_0.9825_O_3_ within *T* = 293–423 K. But in order to compare with the results of Pb_0.89_La_0.11_(Zr_0.7_Ti_0.3_)_0.9725_O_3_, the 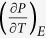 and ECE results of the antiferroelectric at 3 MV/m were also calculated and shown. It is important to note that the lower integration limit is 0 in the calculation of antiferroelectric ECE at 3 MV/m. 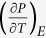 of the two samples at selected electric fields are depicted in Figure S5(a) and (b) in the Supplementary Information. The bulk densities are 7.51 and 7.58 g/cm^3^ for Pb_0.89_La_0.11_(Zr_0.7_Ti_0.3_)_0.9725_O_3_ and Pb_0.93_La_0.07_(Zr_0.82_Ti_0.18_)_0.9825_O_3_, respectively. In addition, in accordance with the results of specific heat capacities (*C*) measured by DSC, the variation of the specific heat capacities between room temperature and *T* = 423 K is very small, thus the specific heat capacities can be considered as a constant, viz. 0.28 and 0.29 J/gK for Pb_0.89_La_0.11_(Zr_0.7_Ti_0.3_)_0.9725_O_3_ and Pb_0.93_La_0.07_(Zr_0.82_Ti_0.18_)_0.9825_O_3_, respectively. Moreover, as comparison, ECE was also calculated by using LGD theory and measured by a high resolution calorimeter. The details of the calculation and measurement are present in the Supplementary Information.

Reversible adiabatic temperature changes (*ΔT*) and entropy changes (*ΔS*), including the measured data and the data calculated by Maxwell relation and LGD theory, are illustrated in Fig. 3(a) and (d). For each sample, the curves of *ΔT* and *ΔS* as a function of temperature are presented. It must be noted that the lattice structure of Pb_0.93_La_0.07_(Zr_0.82_Ti_0.18_)_0.9825_O_3_ around and above the Curie temperature is still not clear, thus the ECE was not calculated above 390 K. Also, for the measured ECE, only the values at 3 MV/m were presented. For relaxor ferroelectrics, the maximum values of *ΔT* and *ΔS* obtained by Maxwell relation are 2.21 K and 1.30 J K^−1^ kg^−1^, respectively, at 423 K and 7 MV/m. Besides, as shown in Fig. 3(a) and (c), another peak with maximum ΔT < 1.5 K and maximum ΔS < 1.1 J K^−1^ kg^−1^ can be observed at around 330 K. Owing to the limitation of the test range, the peak above 423 K does not present, but it can be anticipated. Similarly two ECE peaks have also been reported in several perovskite relaxors^26^,^27^,^28^. The first peak near the Curie temperature is accounted for the slow transition of ferroelectric to paraelectric phase, while the second peak around 423 K is caused by the depolarization of the ceramic^29^,^30^. The ECE calculated from elastic Gibbs free energy that decreases monotonously with temperature. The values are much larger than those obtained by Maxwell relation in the whole test range. The measured values at 3 MV/m which are much larger than those deduced from Maxwell relation at the same electric field do not show a large difference but have a peak value (1.36 K, 1.08 J K^−1^ kg^−1^) at 353 K, above the Curie point. Also, the measured values are smaller than the ECE obtained by LGD theory below 350 K, but are comparable to those above 350 K.

While for antiferroelectric sample, as shown in Fig. 3(b) and (d), the electrocaloric properties deduced from Maxwell relation with maximum *ΔT* = 1.04 K and maximum *ΔS* = 0.67 J K^−1^ kg^−1^ are presented at 414 K. There are also two peaks appeared in the curves. One is a broad peak around 330 K, another one occurs at 414 K. The first broad peak is associated with the change of pyroelectric coefficient 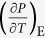 between 293 K and 360 K, as shown in Figure S5(b). In order to further elucidate the phenomena, the P-E loops under 2 and 3 MV/m have been investigated at 333 K, the results are shown in Fig. 4. When the external electric field is 2 MV/m, a typical ferroelectric loop can be observed in Fig. 4(a), but a double hysteresis loop (Fig. 4(b)) is observed at the external electric field of 3 MV/m. As have been verified by XRD analysis (Figure S2) and P-E loop shown in Fig. 2(b), the anti-ferroelectric and ferroelectric phases coexist in the Pb_0.93_La_0.07_(Zr_0.82_Ti_0.18_)_0.9825_O_3_ ceramics. When the electric field is lower than 3 MV/m, the AFE-FE phase transition cannot be induced, thus only the polarization of the ferroelectric phase changes with the electric field and a normal ferroelectric hysteresis loop appears. Due to the two opposite dipoles in one unit cell, the net polarization of antiferroelectric is zero in the pristine state and a large enough electric field is needed to convert the antiferroelectric phase into ferroelectric one (AFE-FE phase transition)^20^. Thus, when the electric field is ≥3 MV/m, the AFE-FE transition is induced following a rapid increase in the polarization. Besides, as the external electric field continues to increase, the growth rate of the polarization is reduced gradually, which can be clearly reflected by the evolution of hysteresis loops shown in Fig. 2(b). Hence this change can be accounted for the 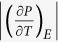 increase as the external electric field is lowered. Similar phenomenon of 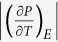 change has been reported by other groups^31^,^32^. Based on above analyses, it can be concluded that the first ECE peak around 330 K may be attributed to the AFE - FE phase transition induced by the electric field in this temperature range. At the same time, the second ECE peak at around 415 K is caused by the AFE - FE phase transition induced by the temperature. Although the ECE occurring at the phase transition temperature can be driven by the temperature or the electric field, it should be noted that the principal reason leading to the ECE is the entropy change resulting from the configuration change of the dipoles under an external electric field. Besides, according to Maxwell relation, negative ECE was observed at 3 MV/m above 410 K, as shown in Fig. 3(b) and (d). The ECEs calculated from LGD theory are much larger than those obtained by the Maxwell relation and even exceed 11.4 K, 8.8 J K^−1^ kg^−1^ at 7 MV/m, respectively. Although the measurements of ECE were not carried out at 5 and 7 MV/m, a strange phenomenon is noteworthy. According to the Maxwell relation, below 3.5 MV/m the ECE values of the antiferroelectric sample are negative at 400–423 K due to the transition of the antiferroelectric to the ferroelectric phase. However, the measured ECEs are quite obvious at this temperature range and even larger than those deduced from Maxwell relation at 7 MV/m during the whole test range. In accordance with the definition of ECE, the adiabatic temperature change is caused by the variation of the ordering degree of dipoles. The grains distribute randomly in the antiferroelectric ceramic, leading to disordered orientation of domains. Thus, as the electric field is applied to the sample, at least a part of the dipoles switches along the field. This behavior gives rise to the increase of the ordering degree of dipoles and ECE.

Furthermore, both of the measured values fluctuated within a narrow range from 303 K to 423 K (0.84–1.36 K for relaxor and 1.05–1.3 K for antiferroelectric), but the variation of ECE values in antiferroelectric ceramics is smaller than those in relaxor ferroelectrics, which may be caused by the synergy of relaxation in the antiferroelectrics.

## Discussion

For the ECE measured directly, the main source of measurement error is in the form of heat that dissipates through objects attached to the sample and surrounding air before reaching the thermocouple. But due to fast internal thermal response times, the method used to obtain the ECE directly in this study has sufficient accuracy^33^. Thus these ECE values are considered to close to the actual situation.

The Maxwell relation has been widely used in deducing the ECE of single crystals or polycrystalline ferroelectric materials^21^,^22^,^23^. However, it is not exactly corrected because the *P(T*) relationship cannot be formulated exactly, but only empirically by fitting the measured *P(T*) dependences at a constant electric field^3^. In addition, in a multi-domain material the domain dynamics under the applied field reduces the excess entropy available for the transfer to acoustic modes and thus reduces the ECE^3^,^34^. To increase the accuracy of the indirect method the system must be a single domain crystal, which can, in the first approximation, be achieved at the state of saturated polarization^3^,^34^. Moreover, the Maxwell relation is derived under the assumption that the thermodynamic system is ergodic^5^,^35^,^36^. Thus, the results obtained by the Maxwell relation are not accurate when applying it to relaxor ferroelectrics, since these material systems are nonergodic. Lu *et al*.^11^ have compared direct and indirect measurements on a PVDF-based relaxor ferroelectric polymer and showed that with the Maxwell relation, the ECE was not satisfactorily reflected and much smaller than the results of direct measurement. Here similar results are obtained.

For the ECE deduced from the LGD theory, in the elastic Gibbs free energy expression, the coefficient *a* was approximately assumed linearly temperature dependent as usual for normal ferroelectrics and *α* equals to (*ε*_0_*C*)^−1^. But according to the results of Pirc *et al*.^37^, in relaxor ferroelectrics, *α* is not constant and expected to be a function of temperature. Moreover, for normal ferroelectrics the paraelectric phase appears instantly above the Curie temperature, while ferroelectric ordering still exists in the entire Curie range in relaxor ferroelectrics due to the persistence of polar nanoregions may above the peak temperature of the permittivity^38^. Hence, large errors will emerge from the calculation of *α* using the LGD formula, Equation S3. In addition, the grain boundaries, the non-ferroelectric grain boundary layers, the domain walls and defect dipoles will have impact on the polarization^39^,^40^,^41^,^42^, and cause errors to the ECE deduced from the Gibbs free energy expression.

The peak values of ECE properties of the two compositions are summarized in Table 2. By comparison, the maximum ECE properties of several FE and AFE bulk ceramics obtained by Maxwell relation and direct measurement are also listed.

For the samples listed in Table 2, the compositions of this work show relatively large values, no matter, whether they were obtained by Maxwell relation or measured directly. Besides, the two samples demonstrate relatively large ECEs in the whole test range, which are better than other samples shown in Table 2. Many recent studies have illustrated that the giant electrocaloric effect has been observed in thin films, including inorganic ceramic thin films and organic films, such as PbZr_0.95_Ti_0.05_O_3_^25^, Pb_0.8_Ba_0.2_ZrO_3_^31^, and poly (vinylidene fluoride-trifluoroethylene) [P(VDF-TrFE)]^23^. For these films, although the reversible adiabatic temperature changes (*ΔT*) are large (*ΔT* > 10 K), their electrocaloric efficiency (*ΔT*/*ΔE*) are not very large^23^,^25^,^31^. For example, the ΔT for PbZr_0.95_Ti_0.05_O_3_ thin film is 12 K, but its electrocaloric efficiency *ΔT*/*ΔE* = 0.15 (10^−6^ K m/V), which is much smaller than our results. The reason is that the film can sustain extremely high electric field. The typical dependence of the dielectric strength (*E*_*b*_) on thickness (*h*) follows the Forlani and Minnaja’s relationship^43^: *E*_*b*_ ∝ *h*^−*n*^, where n is the fitting parameter which depends on the microstructure and the charge transfer properties of the material. For very thin films, which often exhibit lower concentration of structural defect, the electric field is distributed more uniformly over the film with fewer hot spots, thus thin film has a higher breakdown electric field. In general, the situation in thin film is more similar to an ideal crystal lattice which has almost no defect, and the dielectric strength is closer to the theoretical value^44^. The bulk ceramics are limited by their low electric breakdown strength due to the extrinsic factors, e.g., defects, voids, interfaces, etc.

In summary, the ECE of the relaxor ferroelectric Pb_0.89_La_0.11_(Zr_0.7_Ti_0.3_)_0.9725_O_3_ and the antiferroelectric Pb_0.93_La_0.07_(Zr_0.82_Ti_0.18_)_0.9825_O_3_ ceramics are calculated using the Maxwell relation and Landau-Ginzburg-Devonshire (LGD) phenomenological theory and measured by a thermometer and electric field controlled by a computer directly. The microstructures and phase compositions of the samples were also investigated and discussed in connection with the ECE results. The relaxor ferroelectric Pb_0.89_La_0.11_(Zr_0.7_Ti_0.3_)_0.9725_O_3_ and antiferroelectric Pb_0.93_La_0.07_(Zr_0.82_Ti_0.18_)_0.9825_O_3_ ceramics show similarly large electrocaloric efficiency (*ΔT*/*ΔE*) and temperature change by direct measurements. For the antiferroelectric Pb_0.93_La_0.07_(Zr_0.82_Ti_0.18_)_0.9825_O_3_ sample, the ECE deduced from LGD theory is one order of magnitude larger than the results calculated by Maxwell relations and shows peak values around the phase transition temperatures.

## Methods

### Preparation of samples

**Figure 1:**
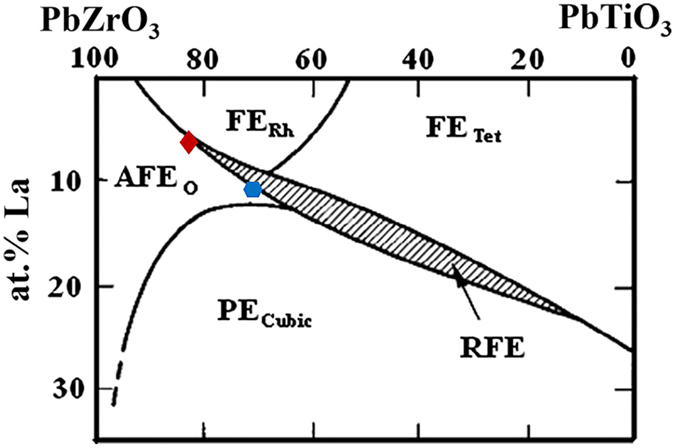
Composition positions of Pb_0.89_La_0.11_(Zr_0.7_Ti_0.3_)_0.9725_O_3_ (right) and Pb_0.93_La_0.07_(Zr_0.82_Ti_0.18_)_0.9825_O_3_ (left) in the room temperature phase diagram of the PLZT system^13^.

**Figure 2:**
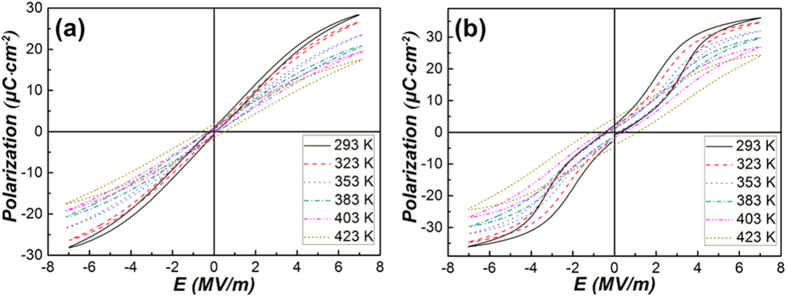
P–E hysteresis loops of Pb_0.89_La_0.11_(Zr_0.7_Ti_0.3_)_0.9725_O_3_ (**a**) and Pb_0.93_La_0.07_(Zr_0.82_Ti_0.18_)_0.9825_O_3_ (**b**) ceramics at different temperatures.

**Figure 3:**
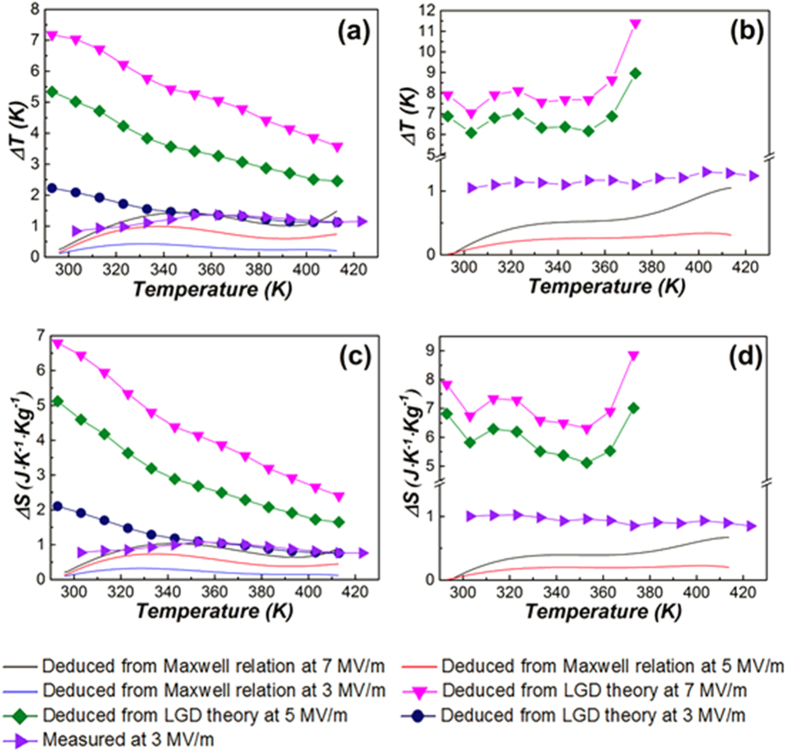
Reversible adiabatic temperature changes (*ΔT*) for Pb_0.89_La_0.11_(Zr_0.7_Ti_0.3_)_0.9725_O_3_ (**a**) and Pb_0.93_La_0.07_(Zr_0.82_Ti_0.18_)_0.9825_O_3_ (**b**) ceramics, reversible adiabatic entropy changes (*ΔS*) for Pb_0.89_La_0.11_(Zr_0.7_Ti_0.3_)_0.9725_O_3_ (**c**) and Pb_0.93_La_0.07_(Zr_0.82_Ti_0.18_)_0.9825_O_3_ (**d**) ceramics. Data labels in (**b**,**d**) are the same with those in (**a**,**c**).

**Figure 4:**
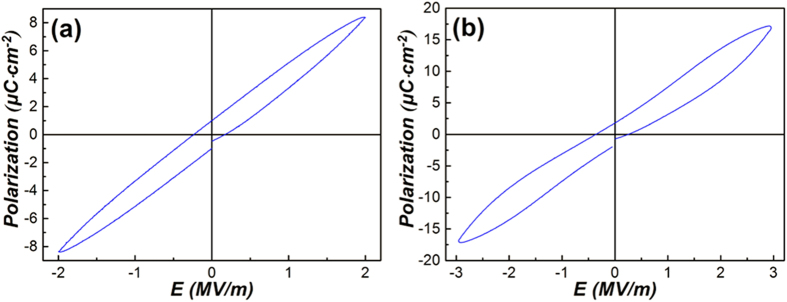
P-E loops of Pb_0.93_La_0.07_(Zr_0.82_Ti_0.18_)_0.9825_O_3_ ceramics under electric field of 2 MV/m (**a**) and 3 MV/m (**b**) at 333 K.

Commercial PbO, La_2_O_3_, TiO_2_ and ZrO_2_ (99.99% in purity, ALADDIN) were used as the raw materials. The batch materials were weighed in accordance with the nominal formulas of Pb_0.89_La_0.11_(Zr_0.7_Ti_0.3_)_0.9725_O_3_ and Pb_0.93_La_0.07_(Zr_0.82_Ti_0.18_)_0.9825_O_3_. Both compositions are located near to the antiferroelectric and relaxor ferroelectric regions in the phase diagram (Fig. 1 13). Pb_0.93_La_0.07_(Zr_0.82_Ti_0.18_)_0.9825_O_3_ is near the ferroelectric, antiferroelectric, and relaxor ferroelectric regions, while Pb_0.89_La_0.11_(Zr_0.7_Ti_0.3_)_0.9725_O_3_ touches the antiferroelectric and relaxor ferroelectric phase boundary. An excess of PbO (2 wt.%) was added to compensate the lead loss during the sintering at high temperatures.

The preparation of ceramics is as follows: firstly, the batch materials were ball milled for 24 h using a planetary mill. After ball milling, the wet mixture was dried and calcined at 900 °C in air for 3 h to form PLZT crystallites. The calcined powders were then ground and particulated with a PVB binder and further pressed into disks with a diameter of 12 mm using a uniaxial pressure of 250 MPa.

The disks were then placed in a muffle furnace and heated up to 600 °C at a rate of 5 °C/min in air and soaked for 3 h to burn out the binder. Then the disks were placed in an alumina crucible and buried in already sintered Pb(Zr_0.5_Ti_0.5_)O_3_ powder to compensate the lead loss. The sintering was carried out in air. In order to obtain fine grains and uniform grain sizes, the samples were heated up at a rate of 10 °C/min and kept at a lower temperature (1100–1140 °C) first for about 4 h, and then fired at a higher temperature (1220–1240 °C) for about 30 min. In addition, during the cooling period between the sintering temperature and 900 °C, a cooling rate of 5 °C/min was used to control the cooling process to reduce the stress between grains. The actual sintering process data for each sample are listed in Table 1.

### Characterization

The ceramic samples were carefully polished and both surfaces of the plates covered with gold as contact electrodes for testing. The density of the samples was measured using the Archimedes method in d.i. water. The polycrystalline structure was characterized using an X-ray diffractometer (XRD, Rigaku D/max-2200PC; Cu Kα radiation, λ = 1.5406 Å). The morphology was observed by a scanning electron microscopy (SEM: JSM-7500). The dielectric constant and loss tangent were measured using an impedance analyzer (Agilent 4284A) at a voltage = 1 V and zero bias field. The polarization - electric field (P-E) hysteresis loop was obtained from a Sawyer-Tower circuit (RADIANT RT66A). The specific heat capacities were tested by a differential scanning calorimeter (Mettler-Toledo DSC-3) in a modulated mode.

## Additional Information

**How to cite this article:** Lu, B. *et al*. Large Electrocaloric Effect in Relaxor Ferroelectric and Antiferroelectric Lanthanum Doped Lead Zirconate Titanate Ceramics. *Sci. Rep.* **7**, 45335; doi: 10.1038/srep45335 (2017).

**Publisher's note:** Springer Nature remains neutral with regard to jurisdictional claims in published maps and institutional affiliations.

## Supplementary Material

Supplementary Information

## Figures and Tables

**Table 1:** Sintering processes for the two samples.

Samples	First heat preservation/time	Second heat preservation/time
Pb_0.89_La_0.11_(Zr_0.7_Ti_0.3_)_0.9725_O_3_	1100 °C/240 min	1225 °C/30 min
Pb_0.93_La_0.07_(Zr_0.82_Ti_0.18_)_0.9825_O_3_	1130 °C/250 min	1230 °C/30 min

**Table 2:** Electrocaloric characteristics of some bulk ceramics.

Material	Measurement method	T (K)	ΔT (K)	ΔE (MV/m)	ΔT/ΔE (10^−6^ K∙m/V)	Refs.
Pb(Mg_0.5_W_0.5_)_0.5_Ti_0.5_O_3_	MR^a^	423	0.3	2.3	0.13	3, 32
Pb(Zr_0.43_Sn_0.43_Ti_0.14_)O_3_	MR/DTR^b^	343	0.27	3	0.09	3, 45
Pb(Zr_0.455_Sn_0.455_Ti_0.09_)O_3_	MR/DTR	319	1.05/1.3	3	0.35/0.43	45
(PbZrO_3_)_0.71_(BaTiO_3_)_0.29_	MR/DTR	298	0.15	2	0.08	3, 45
Pb_0.89_La_0.11_(Zr_0.7_Ti_0.3_)_0.9725_O_3_	MR/DTR	423/353	2.21/1.36	7/3	0.32/0.453	this work
Pb_0.93_La_0.07_(Zr_0.82_Ti_0.18_)_0.9825_O_3_	MR/DTR	414/403	1.04/1.3	3.5/3	0.30/0.433	this work

^a^Maxwell relation; ^b^Direct temperature reading.
