# Supplementary material for: Large Electrocaloric Effect in Relaxor Ferroelectric and Antiferroelectric Lanthanum Doped Lead Zirconate Titanate Ceramics
Source: Sci Rep. 2017 Mar 27;7:45335. doi: 10.1038/srep45335 (PMC5366905; doi:10.1038/srep45335)
Supplement: Supplementary Information [file srep45335-s1.pdf]

Supplementary Information

Large Electrocaloric Effect in Relaxor Ferroelectric and Antiferroelectric Lanthanum Doped Lead Zirconate Titanate Ceramics

Biao Lu, Peilian Li, Zhenghua Tang, Yingbang Yao, Xingsen Gao, Wolfgang Kleemann, Sheng-Guo Lu[*]

SEM images: Figure S1 shows the scanning electron microscopy images of the two samples.

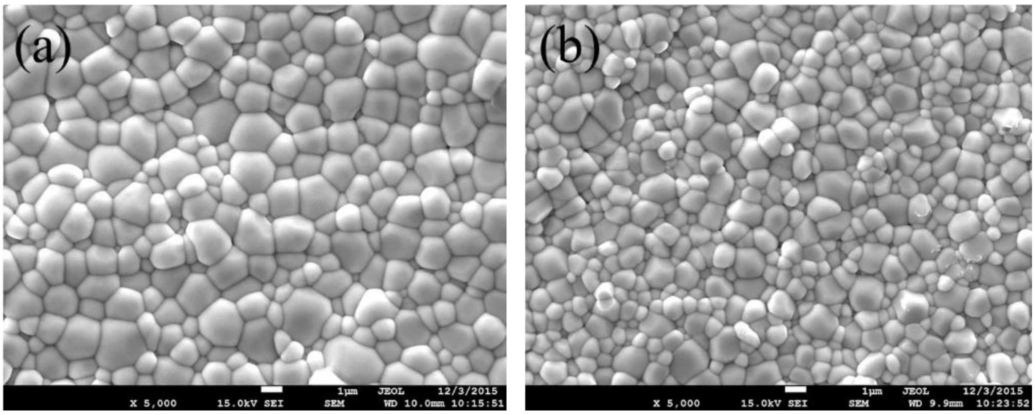

Figure S1 SEM micrographs of $Pb_{0.89}La_{0.11}(Zr_{0.7}Ti_{0.3})_{0.9725}O_3$ (a) and $Pb_{0.93}La_{0.07}(Zr_{0.82}Ti_{0.18})_{0.9825}O_3$ (b) ceramics

XRD Patterns: Figure S2 shows the XRD patterns of two samples, right three panels show the (110), (200) and (222) peaks of two samples.

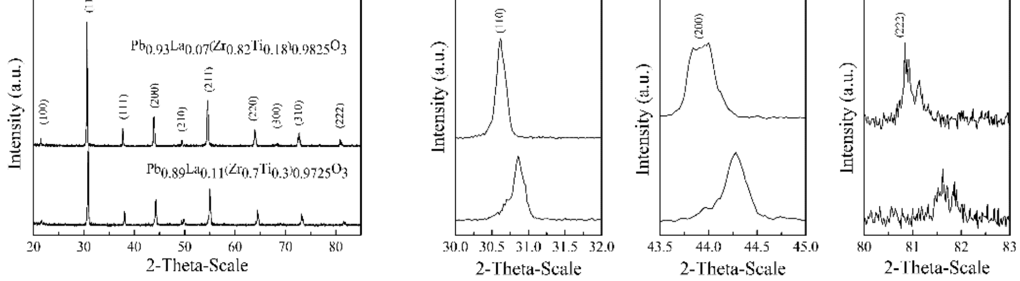

Figure S2 XRD patterns of the PLZT ceramics and extended XRD patterns of (1 1 0), (2 0 0) and (2 2 2) peaks

Dielectric Proprties: Figure S3 exhibits the permitticities as a function of temperature and

frequency, and also the log-log plot of inverse of permittivity ($1/\varepsilon$-$1/\varepsilon_m$) versus temperature (T-T_m).

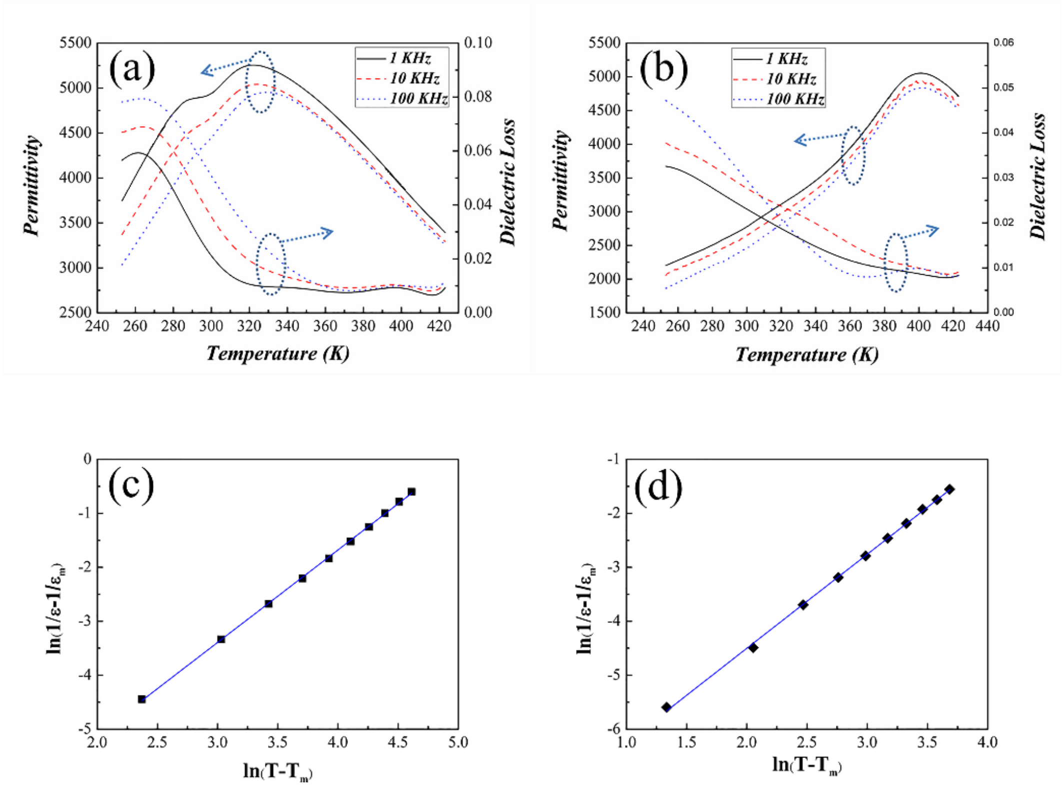

Figure S3 Permittivity and dielectric loss as a function of temperature for $Pb_{0.89}La_{0.11}(Zr_{0.7}Ti_{0.3})_{0.9725}O_3$ (a) and $Pb_{0.93}La_{0.07}(Zr_{0.82}Ti_{0.18})_{0.9825}O_3$ (b) ceramics, logarithmic plots of the reciprocal permittivity $(\frac{1}{\varepsilon}-\frac{1}{\varepsilon_m})$ (measured at 1 kHz) vs. temperature ($T-T_m$) relation for $Pb_{0.89}La_{0.11}(Zr_{0.7}Ti_{0.3})_{0.9725}O_3$ (c) and $Pb_{0.93}La_{0.07}(Zr_{0.82}Ti_{0.18})_{0.9825}O_3$ (d) ceramics

Polarization properties: Figure S4 shows the polarization as a function of temperature and external electric field for two samples.

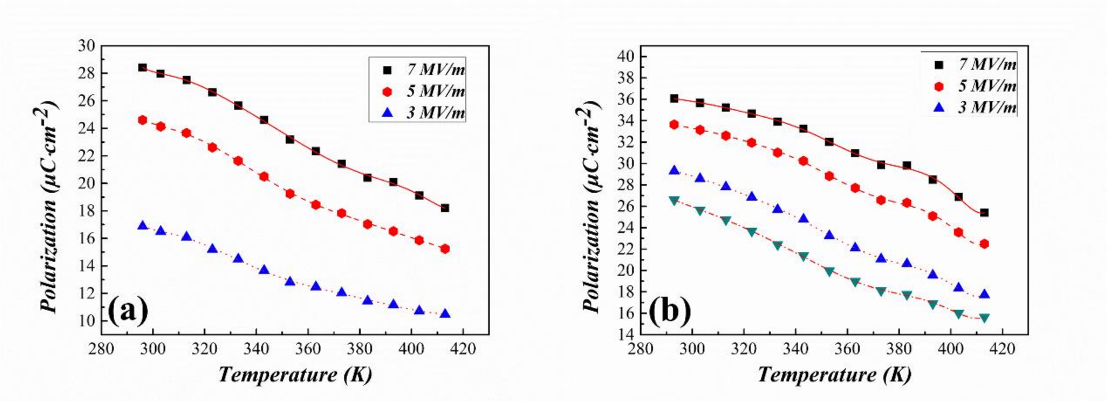

Figure S4 Temperature dependence of the polarization for $Pb_{0.89}La_{0.11}(Zr_{0.7}Ti_{0.3})_{0.9725}O_3$ (a) and $Pb_{0.93}La_{0.07}(Zr_{0.82}Ti_{0.18})_{0.9825}O_3$ (b) ceramics

Pyroelectric properties: Figure S5 shows the pyroelectric coefficient (dP/dT) as a function of temperature and external electric field for two samples.

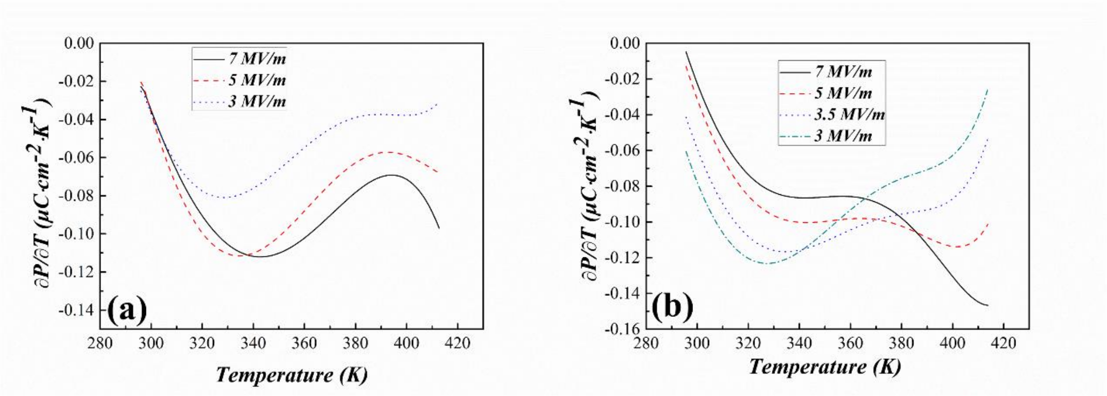

Figure S5 $\left(\frac{\partial P}{\partial T}\right)_E$ at selected electric fields for $Pb_{0.89}La_{0.11}(Zr_{0.7}Ti_{0.3})_{0.9725}O_3$ (a) and $Pb_{0.93}La_{0.07}(Zr_{0.82}Ti_{0.18})_{0.9825}O_3$ (b) ceramics

Relaxor ferroelectric properties: In general, the relaxation behavior of ferroelectric can be determined by the modified Curie-Weiss law [S1]

$$\frac{1}{\varepsilon} - \frac{1}{\varepsilon_m} = \frac{(T - T_m)^\gamma}{C'} \quad \text{(S1)}$$

where ε_m and T_m are the maximum dielectric constant and the corresponding temperature, ε and T the dielectric constant and corresponding temperature above T_m, C' the Curie-like constant. γ is the critical exponent and associated with the type of ferroelectric. When $\gamma = 1$ and 2, the material is corresponding to an ideal normal ferroelectric and to an ideal relaxor ferroelectric, respectively. The relaxation behavior of the ferroelectric is gradually increasing with γ when γ is between 1 and 2. γ can be worked out by fitting the logarithmic plots of the reciprocal permittivity $(\frac{1}{\varepsilon} - \frac{1}{\varepsilon_m})$ measured at the same frequency as a function of temperature $(T - T_m)$. As shown in Figures S3 (c) and (d), the linearities of the curves are very good. γ has been determined from the slope of the straight line: $\gamma = 1.71$ for $Pb_{0.89}La_{0.11}(Zr_{0.7}Ti_{0.3})_{0.9725}O_3$ and 1.74 for $Pb_{0.93}La_{0.07}(Zr_{0.82}Ti_{0.18})_{0.9825}O_3$.

Phenomenological calculation: For relaxor ferroelectrics, the crystalline phase has a pseudo-cubic structure. The elastic Gibbs free energy can be expressed as follows [S2]

$$F = F_0 + \frac{1}{2}aP^2 + \frac{1}{4}bP^4 + \frac{1}{6}cP^6 + \cdots - EP \qquad \text{(S2)}$$

where b and c are assumed to be temperature-independent phenomenological coefficients. For the parameter a a linear temperature dependence based on the Curie-Weiss law [S3],

$$a(T) = \alpha(T - T_0), \qquad \text{(S3)}$$

approximately holds. The coefficient $\alpha = (\varepsilon_0 C)^{-1}$ is related to the Curie constant C. T_0 is the Curie-Weiss temperature and can be obtained by fitting the inverse of the dielectric constant versus temperature in the paraelectric state above T_0 using the Curie-Weiss law. Then the ECE can be obtained by the following relations

$$\Delta S = -\left(\frac{\partial F}{\partial T}\right)_P = -\frac{1}{2}\alpha P^2, \qquad \text{(S4)}$$

$$\Delta T = {}^{T\Delta S}/_{C}. \qquad \text{(S5)}$$

Due to the lack of a $Pb_{0.89}La_{0.11}(Zr_{0.7}Ti_{0.3})_{0.9725}O_3$ single crystal, the dielectric data of $Pb_{0.89}La_{0.11}(Zr_{0.7}Ti_{0.3})_{0.9725}O_3$ ceramic were measured, which are supposed to yield similar values of T_0 and C as single crystals[S3].

The Landau-Ginzburg-Devonshire (LGD) phenomenological theory has also been used to explain the phase transition and dielectric properties of the antiferroelectric PZT system[S3-S5]. For the antiferroelectric with orthorhombic symmetry, the polarization is along the [110] direction. The Gibbs free energy of antiferroelectrics orthorhombic (A_O) phase for the PZT system under zero stress conditions can be described as follows [S4]

$$\Delta G(\mathrm{O}) = 2\sigma_1 p_3^2 + (2\sigma_{11} + \sigma_{12})p_3^4 + 2(\sigma_{111} + \sigma_{112})p_3^6 \ , \qquad \text{(S6)}$$

where, p_i, σ_1, σ_{ij}, and σ_{ijk} (i, j, k=1, 2, 3) denote the polarization components along the coordinate axis, and antiferroelectric dielectric stiffness at a constant stress, respectively.

It should be noted that the above relations are merely suitable for antiferroelectric single domains[S4]. Based on them, the single-domain properties of PLZT can be determined and the intrinsic contributions to the properties understood. Hence, by neglecting extrinsic contributions (e.g. domain wall and defect motions), the theories can be used to further understand the properties of polycrystalline materials[S4].

For the antiferroelectric ceramics, the grains distribute randomly, which leads to disordered orientation of domains. When an electric field is applied on the polycrystalline ferroelectric ceramic, the distortions of at least some of the crystallites, initially randomly distribute, orient along the allowable direction along the poling electric field. Some literatures have reported the polarization of ferroelectric ceramics and crystals with the same composition at the same poling condition[S6,S7]. The relationship between upper limits $\bar{P}$ of the polarization of the ceramic and P of the antiferroelectric/ferroelectric single-domain is as follows[S7]: tetragonal ceramic $\bar{P}$=0.831 P, rhombohedral ceramic $\bar{P}$=0.866 P, and orthorhombic ceramic $\bar{P}$=0.912 P. All of the coefficients of the Gibbs free energy function were independent of temperature, except for the antiferroelectric and ferroelectric dielectric stiffness coefficients σ_1 and α_1, which were given as linear temperature dependences based on the Curie-Weiss law[S3,S7,S8]. For the antiferroelectric orthorhombic phase, let σ_1 be $\beta(T\text{-}T_C)$. Further, β, $2\sigma_{11}+\sigma_{12}$, and $\sigma_{111}+\sigma_{112}$ in the equation (S6) can be found from the first partial derivative stability conditions:

$$E_3 = \left(\frac{\partial \Delta G}{\partial p_{S3}}\right)_T = 4\beta\,(T - T_C)p_{S3} + 4\,(2\sigma_{11} + \sigma_{12})\,p_{S3}^3 + 12\,(\sigma_{111} + \sigma_{112})\,p_{S3}^5, \qquad \text{(S7)}$$

where E_3 and p_{S3} are the electric field and the polarization components of a single-domain material along the coordinate axis. The electric field strengths, 5, 6 and 7 MV/m and their corresponding polarizations were selected respectively and substituted into Equation (S7) to procure the coefficient β. Then the reversible adiabatic changes in entropy (ΔS) and temperature (ΔT) can be obtained by using the relations as mentioned in Equations (S4) and (S5), and the polarization p_{C3} as well.

The parameters (α) used for the calculation of electrocaloric effect are listed in the Table S1.

Table S1. α values of the samles

Material	α (×10^5 K^{-1})								
	293K	303K	313K	323K	333K	343K	353K	363K	373K
$Pb_{0.89}La_{0.11}(Zr_{0.7}Ti_{0.3})_{0.9725}O_3$	11.9 (between 293 K and 413 K)								
$Pb_{0.93}La_{0.07}(Zr_{0.82}Ti_{0.18})_{0.9825}O_3$	9.12	8.02	8.96	9.19	8.67	8.89	9.32	10.9	15.03

Direct ECE measurement: For the direct measurement, a thermometer and an electric field controlled by a computer were employed to detect the temperature change caused by ECE as the application or withdrawing of an electric field. Also, a high-voltage generator controlled by an arbitrary signal generator is used to generate the electric field step signal, which is then applied to the sample. The voltage should be maintained for a few second, in order to get into thermal equilibrium with the surrounding. Then the voltage was released immediately. The typical thermal response times along the sample thickness direction is a few milliseconds. Within such a short period, a very fast equilibration of the temperature throughout the whole sample, including the electrodes, attached thermometer and wires, took place, but then the equilibrated sample exchanges the heat on a much longer time scale to the surrounding bath (T_{bath}). So the relaxation of the temperature of the whole system can be described as [S9]

$$T(t) = T_{bath} + \Delta T e^{-\frac{t}{\tau}} \ , \qquad \text{(S8)}$$

where T_{bath} is the surrounding temperature and t the heat transfer time. More details about the test procedure and data analysis can be found in Ref. S9 and S10.

During this test, an electric field of 3 MV/m was applied to the sample for 15 seconds to obtain temperature equilibrium first, then the electric field was released immediately. Meanwhile, the ECE signal appears as shown in Figure S6. The red curves are the fitted curves using equation (S8). ΔT_{ECE} is obtained by extrapolating the fitting toward the time of the fall of the step-like pulse. ΔT_{ECE} is measured in the temperature range from 303 K to 423 K at successive increments of 10 K in the temperature range of 303 K to 423 K. In the direct measurement of ΔT, one concern is the Joule heating in the samples, which will cause the enhancement of temperature when the field is applied. But in this test, the base line temperature T in Figure S6 is constant except while withdrawing the electric field, which indicates that the observed temperature change is due to ECE.

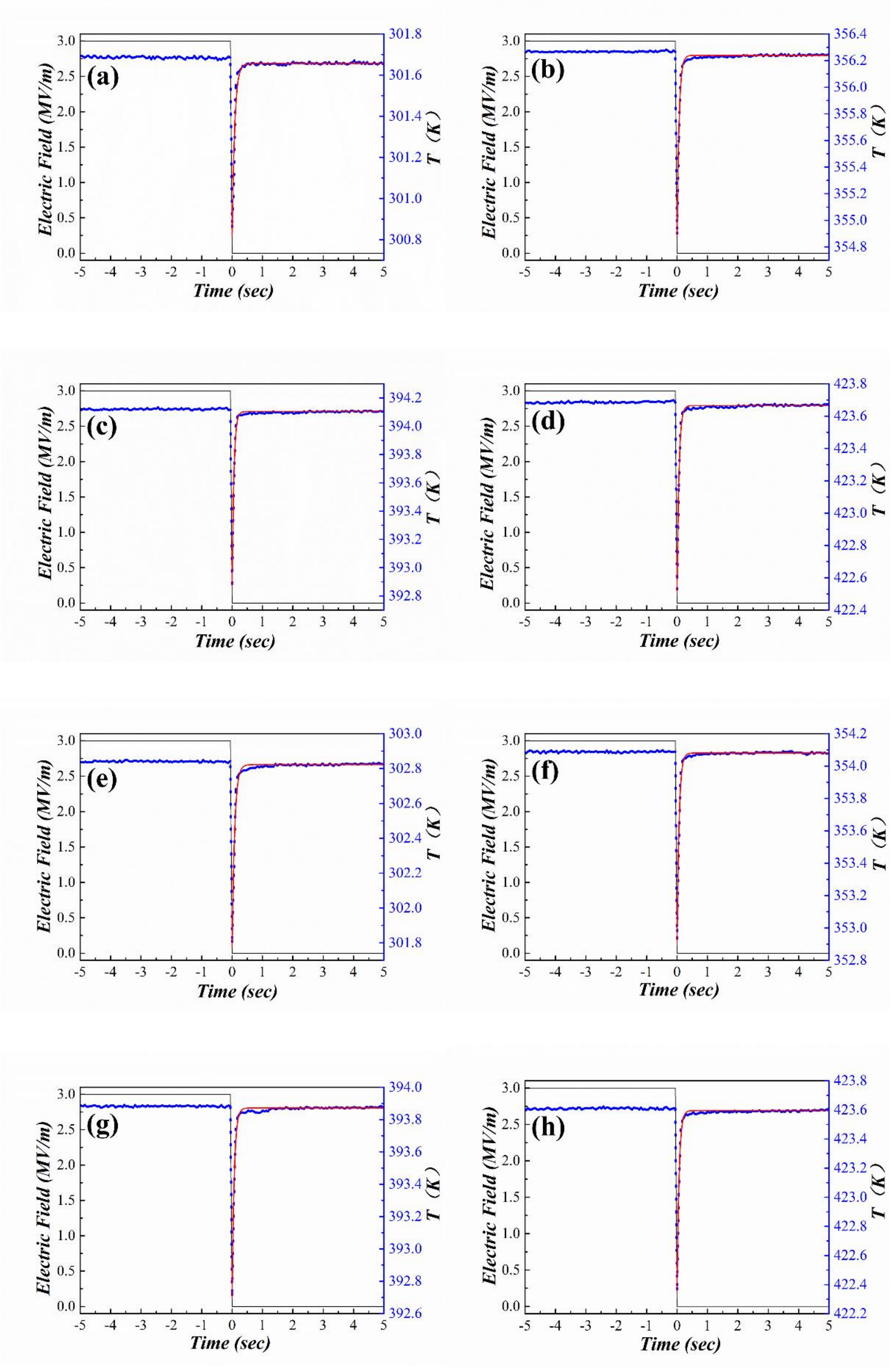

Figure S6 Representative measured values for $Pb_{0.89}La_{0.11}(Zr_{0.7}Ti_{0.3})_{0.9725}O_3$ at 301 K (a), 356 K (b), 393 K (c), 423 K (d) and for $Pb_{0.93}La_{0.07}(Zr_{0.82}Ti_{0.18})_{0.9825}O_3$ at 302 K (e), 354 K (f), 393 K (g), 423 K (h) with the electric field of 3MV/m. The short blue dash line is the measured data. The red line

is the fitting curve.

References

S1. Bokov, A. A. *et al.* Dielectric relaxation in relaxor ferroelectrics. *J. Adv. Dielect.* **2,** 1241010 (2012).

S2. Pirc, R. *et al.* Electrocaloric effect in relaxor ferroelectrics. *J. Appl. Phys.* **110,** 074113(2011).

S3. Haun, M. J. *et al.* Thermodynamic theory of the lead zirconate-titanate solid solution system, part II: Tricritical behavior. *Ferroelectrics* **99,** 27-44(1989).

S4. Haun, M. J. *et al.* Thermodynamic theory of the lead zirconate-titanate solid solution system, part I: phenomenology. *Ferroelectrics* **99,** 13-25(1989).

S5. Haun, M. J. *et al.* Thermodynamic theory of the lead zirconate-titanate solid solution system, part III: Curie constant and sixth-order polarization interaction dielectric stiffness coefficients. *Ferroelectrics* **99,** 45-54(1989).

S6. Berlincourt, D. *et al.* Domain processes in lead titanate zirconate and barium titanate ceramics. *J. Appl. Phys.* **30,** 1804-1810 (1959).

S7. Baerwald, H. G. Thermodynamic theory of ferroelectric ceramics. *Phys. Rev.* **105,** 480(1957).

S8. Baerwald H. G. *et al.* Electromechanical response and dielectric loss of prepolarized barium titanate under maintained electric bias. Part I. *J. Acoust. Soc. Am.* **25,** 703-710(1953).

S9. Yao, H. et al. Nonadiabatic scanning calorimeter. *Rev. Sci. Instrum.* **69,** 172(1998).

S10. Z. Kutnjak, B. Rožič. Indirect and direct measurements of the electrocaloric effect in electrocaloric materials, ed. T. Correia and Q. Zhang, Springer, Berlin Heidelberg, 2014, p. 147-182.
